# Ultrasound-Guided Quadratus Lumborum Block: An Updated Review of Anatomy and Techniques

**DOI:** 10.1155/2017/2752876

**Published:** 2017-01-03

**Authors:** Hironobu Ueshima, Hiroshi Otake, Jui-An Lin

**Affiliations:** ^1^Department of Anesthesiology, Showa University Hospital, Tokyo, Japan; ^2^Department of Anesthesiology, Wan Fang Hospital, Taipei Medical University and Department of Anesthesiology, School of Medicine, College of Medicine, Taipei Medical University, Taipei, Taiwan

## Abstract

*Purpose of Review*. Since the original publication on the quadratus lumborum (QL) block, the technique has evolved significantly during the last decade. This review highlights recent advances in various approaches for administering the QL block and proposes directions for future research.* Recent Findings*. The QL block findings continue to become clearer. We now understand that the QL block has several approach methods (anterior, lateral, posterior, and intramuscular) and the spread of local anesthetic varies with each approach. In particular, dye injected using the anterior QL block approach spread to the L1, L2, and L3 nerve roots and within psoas major and QL muscles.* Summary*. The QL block is an effective analgesic tool for abdominal surgery. However, the best approach is yet to be determined. Therefore, the anesthetic spread of the several QL blocks must be made clear.

## 1. Introduction

The quadratus lumborum (QL) block was first described by Blanco [1]. Currently, the QL block is performed as one of the perioperative pain management procedures for all generations (pediatrics, pregnant, and adult) undergoing abdominal surgery [2–4]. However, disagreement regarding the best approach for administering the block prevails because of unclear mechanisms responsible for the effects and complicated nomenclature system.

## 2. Ultrasound Identification of QL

After recognizing three layers of abdominal wall muscles, transversus abdominis is traced more posteriorly until the transversus aponeurosis appears. At this region, usually we can find the peritoneum curves away from the muscles from anterior to posterior and the retroperitoneal fat lies behind the peritoneum and deep to the transversalis fascia. The retroperitoneal fat is generally scanty above the iliac crest and more prominent closer to the iliac crest. Tilting the probe slightly caudal into the pelvis thus improves the view of the retroperitoneal fat and the tapered end of transversus aponeurosis. QL is usually identified medial to the aponeurosis of transversus abdominis muscle [5].

## 3. Nomenclature (Figure 1)

Current literature on the QL block reports 4 different approaches, with authors using varying nomenclature for describing each block. The QL block was first described as an ultrasound-guide “posterior” transversus abdominis plane (TAP) block by Blanco in 2007, approximating the double-pop TAP technique at the lumbar triangle of Petit [1, 6]. However, QL and TAP blocks are essentially different because a posterior TAP block is, by definition, superficial to the TAP and its aponeurosis. In a recent open forum discussion by Blanco, the QL1 block is actually deep to the transversus abdominis aponeurosis [7]. For QL2 block, the injection is posterior to the QL muscle. Furthermore, the QL block described by Børglum et al. was a transmuscular QL block [8], where the local anesthetic is injected anteriorly between the psoas major (PM) muscle and the QL muscle. Finally, for the intramuscular QL block, the local anesthetic is injected directly into the QL muscle [9, 10].

We need to know the anatomy of the tissue layers surrounding the QL muscle, particularly the thoracolumbar fascia (TLF, Figure 2), to understand these QL blocks [11]. The TLF is a sheet of fused aponeuroses and fascial layers that encases the muscles of the back extending from the thoracic to the lumbar spine and affects the spread of local anesthetic [12]. The TLF is divided into 3 layers (anterior, middle, and posterior) around the muscles of the back. The anterior layer is anterior to the QL muscle. The middle layer is located between the erector spinae and the QL muscle. The posterior layer of thoracolumbar fascia encloses the erector spinae instead of the QL muscle. The anterior layer also blends medially with the fascia of the PM and blends laterally with the transversalis fascia. Injection between the anterior layer and QL can spread cranially under the lateral arcuate ligament to the endothoracic fascia and reach the lower thoracic paravertebral space posterior to the endothoracic fascia [13]. As for QL2 block, recently it was disclosed that in the area where the middle lumbar fascia joins the deep lamina of the posterior layer (paraspinal retinacular sheath) on the lateral border of the erector spinae, a triangular structure named the lumbar interfascial triangle (LIFT) was targeted as the optimal point of injection for QL2 block [14]. Not only serving as the conduit for local anesthetic spread into the thoracic paravertebral space, TLF per se with a high-density network of sympathetic fibers as well as mechanoreceptors was also believed to be another main component responsible for the effects of QL block.

It is logically and communicationally easier to name QL blocks based on the needle tip position in relation to QL than the publication sequence or needle trajectory [12]. Accordingly, the QL 1 block is referred to as the lateral QL block because it involves injecting local anesthetic lateral to the QL muscle with the spread at the junction of QL with transversalis fascia, similar to the pattern of transversalis fascia plane block [5]. By the same rule, the QL 2 block is considered a posterior QL block. The transmuscular QL block is named the anterior QL block because it involves injecting the local anesthetic at the anterior aspect of the QL muscle. Finally, the intramuscular QL block is referred to as the intramuscular QL block.

## 4. Techniques of QL Block

### 4.1. Anterior QL Block

The patient was in the lateral position. A low-frequency convex probe was vertically attached above the iliac crest (Figure 3), and a needle was inserted in the plane from the posterior edge of the convex probe through the QL in an anteromedial direction (Figure 4(a)). The needle tip was placed between the PM muscle and the QL muscle and the local anesthetic was injected into the fascial plane. We confirmed that the local anesthetic appeared to press down the PM in the ultrasound image (Figure 4(b)).

Also, there is an another anterior QL block with paramedian sagittal oblique (subcostal) approach (subcostal QL block) [13]. The patient was in the lateral position. A low-frequency convex probe is placed with a transverse, oblique, and paramedian orientation approximately 3 cm lateral to the L2 spinous process (Figure 5). The needle is then inserted in-plane from the medial side of the transducer and advanced laterally to enter the interfascial plane between the quadratus lumborum and psoas major muscles (Figure 6). With this approach, we think that the psoas major muscle provides a better protective barrier against accidental needle entry into the peritoneal cavity than the thin transversalis fascial layer.

### 4.2. Lateral QL Block

The patient was in the supine position. A high-frequency linear probe was attached in the area of the triangle of Petit (Figure 7) until the QL was confirmed (Figure 8(a)). The needle tip was placed at the anterolateral border of the QL at its junction of QL with transversalis fascia, and the local anesthetic was injected. We confirmed via ultrasound that the local anesthetic is deep to the transversus abdominis aponeurosis (Figure 8(b)).

### 4.3. Posterior QL Block

The patient was in the same supine position as the lateral QL block (Figure 7). The patient was occasionally supported on a pillow to create space under the patient's back to be able to move a low-frequency convex probe freely. The posterior aspect of the QL muscle was confirmed, and the needle tip was inserted into this aspect of the QL muscle (Figure 9(a)). The local anesthetic was then injected into the LIFT behind the QL muscle (Figure 9(b)).

### 4.4. Intramuscular QL Block

The patient was also in the same supine position as the lateral QL block (Figure 7), and a high-frequency linear probe was placed slightly cephalad to the iliac crest. The needle tip was advanced until it penetrated the fascia and was inserted into the QL muscle (Figure 10(a)). Test injection was initially administered to verify that the local anesthetic spreads within the QL muscle (Figure 10(b)). Finally, the local anesthetic spread reaching any area between the fascia and the muscle will predict a successful block [10] (Figure 10(c)).

**Figure 1:**
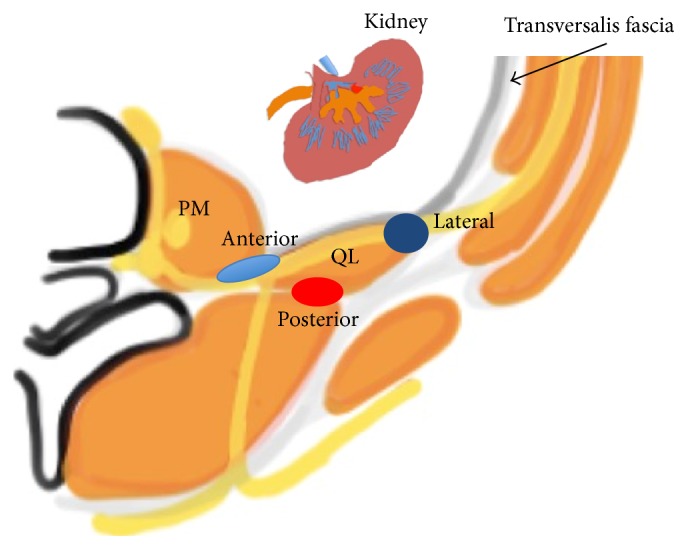
Anatomic view of quadratus lumborum (QL) block (anterior, lateral, and posterior). The lateral QL block injects the local anesthetic at the lateral to the QL muscle. The posterior QL block injects the local anesthetic at the posterior to the QL muscle. The anterior QL block injected the local anesthetic between the PM muscle and the QL muscle. QL: quadratus lumborum muscle, PM: psoas major muscle, and gray line: transversalis fascia.

**Figure 2:**
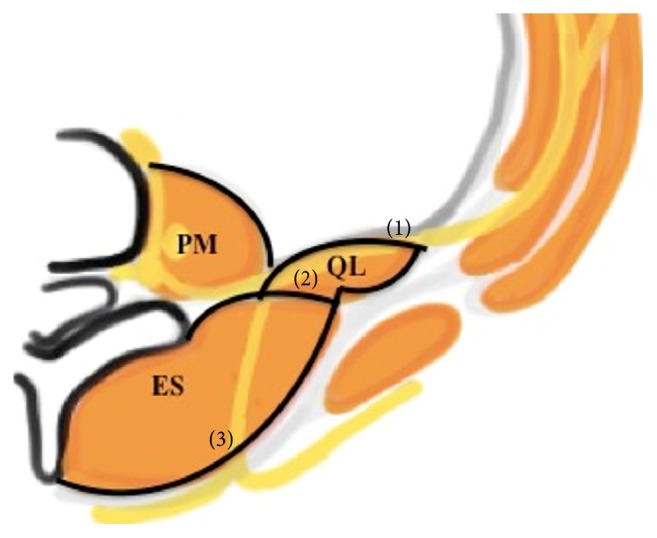
Anatomic view of the thoracolumbar fascia (TLF). The TLF is divided into 3 layers (anterior (1), middle (2), and posterior (3)). QL: quadratus lumborum, ES: erector spinae, LD: latissimus dorsi, and PM: psoas major.

**Figure 3:**
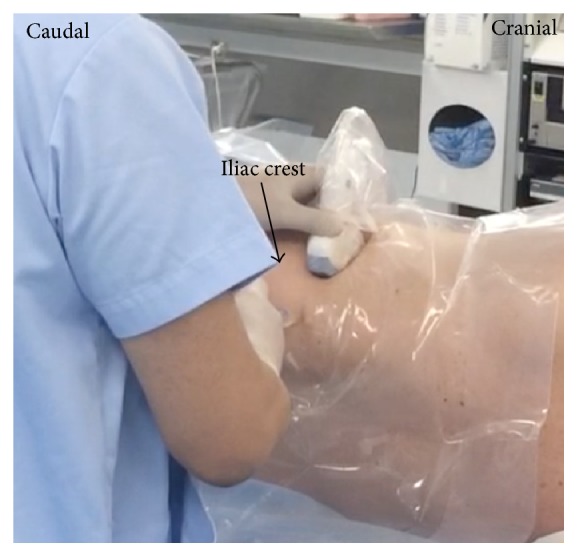
Probe position for anterior QLB. The convex probe was vertically attached above the iliac crest.

**Figure 4:**
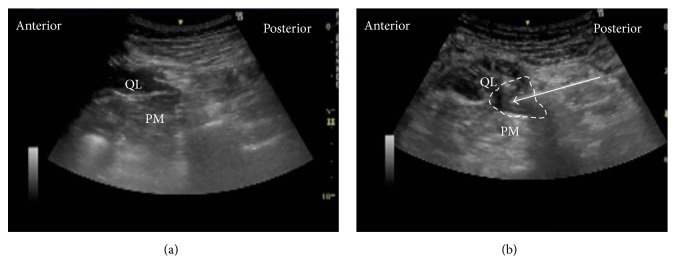
Ultrasound images of anterior QLB. (a) Preinjection and (b) postinjection. QL: quadratus lumborum, PM: psoas muscle, white arrow: needle trajectory, and white dotted line: spread of local anesthetic.

**Figure 5:**
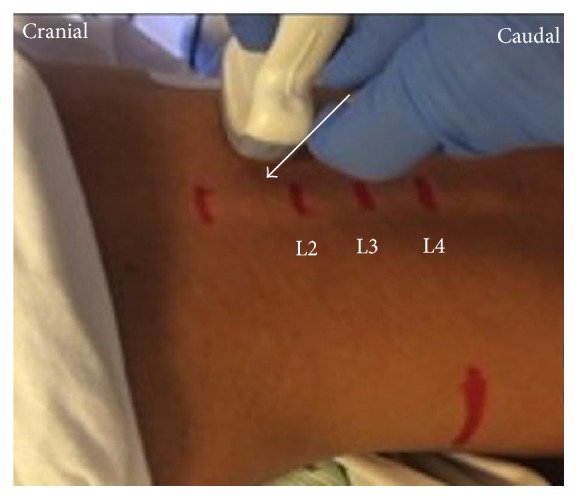
Probe position for subcostal QL block. A low-frequency convex probe is placed with a transverse, oblique, and paramedian orientation approximately 3 cm lateral to the L2 spinous process.

**Figure 6:**
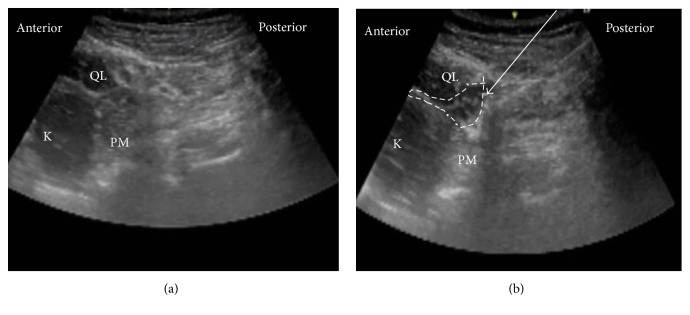
Ultrasound images of subcostal QL block. (a) Preinjection and (b) postinjection. QL: quadratus lumborum, PM: psoas muscle, white arrow: needle trajectory, and white dotted line: spread of local anesthetic.

**Figure 7:**
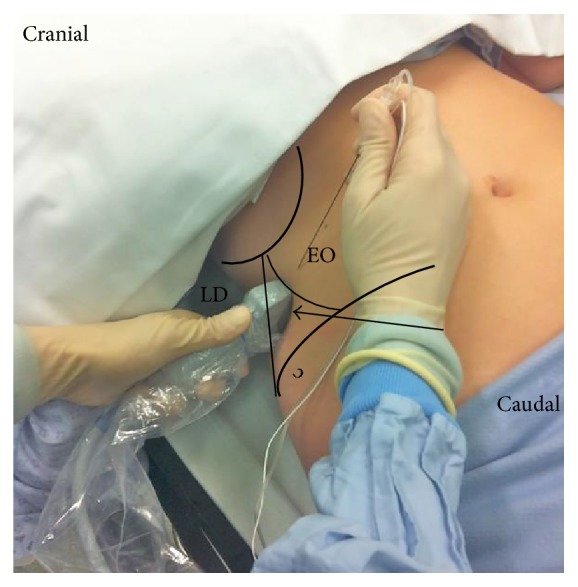
Lateral QL block. A high-frequency linear probe was attached in the area of the triangle of Petit. EO: external abdominal oblique; LD: latissimus dorsi; black arrow: the triangle of Petit.

**Figure 8:**
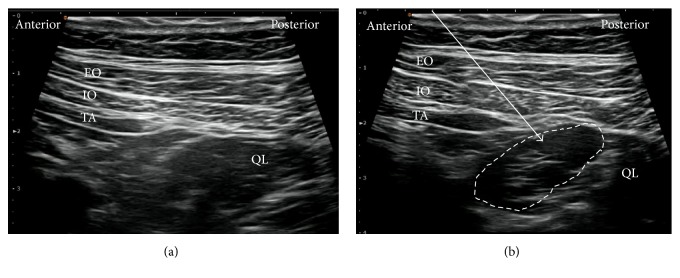
Ultrasound images of lateral QLB. (a) Preinjection and (b) postinjection. EO: external oblique muscle, IO: internal oblique muscle, TA: transversus abdominis, QL: quadratus lumborum, white arrow: needle trajectory, and white dotted line: spread of local anesthetic.

**Figure 9:**
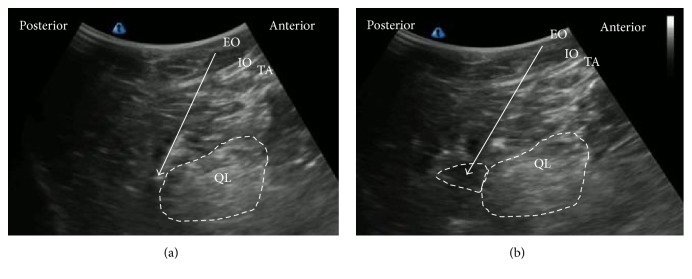
Ultrasound images of posterior QLB. (a) Preinjection and (b) postinjection. EO: external oblique muscle, IO: internal oblique muscle, TA: transversus abdominis, QL: quadratus lumborum, white arrow: needle trajectory, and white dotted line: spread of local anesthetic.

**Figure 10:**
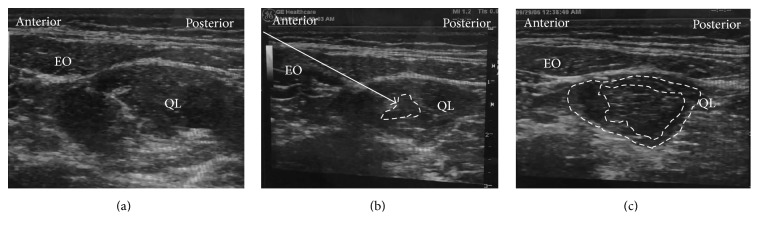
Ultrasound images of intramuscular QLB. (a) Preinjection, (b) test injection, and (c) postinjection. EO: external oblique muscle, QL: quadratus lumborum, white arrow: needle trajectory, and white dotted line: spread of local anesthetic within (b) or in between (c).

We also recommend adding pressure monitors to avoid possible intrafascicular spread during administration of these blocks [15]. This is especially important for anterior and lateral QL blocks, because nerves are located anterior to the QL where the needle tip will be placed (Figure 1). Another reason for adding the half-the-air pressure monitor is to reduce the risk of local anesthetic systemic toxicity (LAST) and at the same time save the local anesthetic when the block site is deep with rich vascularity [16] and needs test injection to confirm the correct spread in the interfascial plane [17], such as the deep anterior QL block involving the thoracolumbar fascia through which vessels exit from the paravertebral space [14].

## 5. Spread of QL Block

A MRI investigation comparing the posterior QL block and the lateral QL block showed that the posterior QL block had spread more than the lateral QL block. Further, the posterior QL block provided a more predictable spread of the local anesthetic into the paravertebral space [3]. However, the calculated volume reaching the paravertebral space was still too small for QL2 block; thus the role of spread into the thoracolumbar plane was considered another synergistic pathway to achieve the effect [14].

Carline et al. investigated the spread of the dye and nerve involvement after 4 anterior, 3 lateral, and 3 posterior QL blocks using an ultrasound-guided technique in soft embalmed cadavers [18]. They injected 20 ml of dye solution for each QL block. The anterior QL block consistently dyed lumbar nerve roots and sometimes nerves within the TAP. The posterior and lateral QL blocks nearly dyed within the TAP, the subcutaneous tissue surrounding the abdominal flank, and into the deep muscles of the back. However the results (especially from the posterior and lateral QL blocks) lack credibility because they were only performed on a few soft embalmed cadavers.

There is no study reporting the dye spread in the intramuscular QL block. Because the injection is intramuscular, the local anesthetic may stay within the QL muscle. Watanabe et al. reported a case undergoing the intramuscular QL block where the spread of the local anesthetic was confirmed by using a fluoroscopy. According to this case, 15 mL of the total radiocontrast injected into the QL muscle remained within the QL muscle [19].

To better assess the mechanism of the several QL blocks, we must simultaneously perform the dye spread study of the 4 different approaches in many soft embalmed cadavers.

## 6. Analgesia (Table 1)

Both the needle trajectory and needle tip position are deemed relevant regarding the spread of local anesthetic after different approaches of QL blocks [12]; thus it is of paramount importance to compare and analyze their analgesic levels, respectively.

The lateral and posterior QL blocks may play a role in conventional perioperative pain management for abdominal surgery [3, 18]. Because the local anesthetic injected via the approach of the posterior QL block can more easily extend beyond the TAP to the thoracic paravertebral space or the thoracolumbar plane [3, 14], the posterior QL block entails a broader sensory-level analgesic than the lateral QL block. Some clinical case studies of patients with caesarean section, gastrostomy, laparoscopy, colostomy, pyeloplasty, and myocutaneous flap surgery showed that the lateral and posterior QL blocks may generate analgesia from T7 to L1 [2–4, 9, 10, 19–22].

For the anterior QL block, the local anesthetic is injected between the PM muscle and the QL muscle. Considering the branches of lumbar plexus nerves run between the PM and the QL, the anterior QLB may play a role in analgesia not only for the trunk but for the lower extremities as well [23]. A dye injection study showed that the anterior QL block consistently dyed lumbar nerve roots and sometimes nerves within the TAP. Therefore, the anterior QL block may generate analgesia from T10 to L4 [18]. For the subcostal QL block (subtype of anterior QL block), the local anesthetic injected anterior to the QL between the QL muscle and the anterior layer of the thoracolumbar fascia observed the spread in cephalad direction close to the T12 rib with anterior displacement of the anterior layer of thoracolumbar fascia. This produces reliable dermatomal coverage from T6-T7 to L1-2 [13].

The intramuscular QL block [9, 10], which involves injection of the local anesthetic directly into the QL muscle, has recently been disclosed. Murouchi et al. reported that, after the lateral QL block, the sensory effects evaluated using a cold test may demonstrate analgesia from T7 to T12 [9]. We consider that the intramuscular QL block is an effective block for lower abdominal surgery such as laparoscopy and femoral-femoral bypass [19].

## 7. Discussion

Since the first description of the QL block about 10 years ago [1], several QL blocks have been reported [1, 7–10, 13, 14]. The QL block cannot generate anesthesia without additional procedures. We do not know whether each QL block relieves complete somatic and visceral pain; hence we recommended QL block as an add-on block to reduce the requirement of general anesthetic intraoperatively or it could be used as the main component of multimodal analgesia postoperatively. Though the anterior and posterior QL blocks may spread the local anesthetic into the paravertebral space, the full scope of spread from each of the four blocks is not clear [3, 18]. To further understand the mechanisms of the several QL blocks, we must perform dye injection study simultaneously using each approach in many soft embalmed cadavers and assess the spread using multiple modalities.

Variable volumes of local anesthetic in regard to each QL block were reported. We are unsure regarding the adequate volume needed to accomplish the block. However, considering previous reports [9, 18], at least 20 mL of the local anesthetic at one site may be required. Because of the large volume, it is important to confirm the safety of the block to avoid LAST. Murouchi et al. measured the local anesthetic concentration after the intramuscular QL block [9]. A total of 150 mg of ropivacaine (0.375%, 20 mL per side) was administered bilaterally. After administration, arterial ropivacaine levels were measured using high-performance liquid chromatography with carbamazepine [9]. The ropivacaine concentration was less than 2.2 *μ*g/mL, which represented the arterial and venous threshold values of systemic toxicity [24]. Therefore, the injection of the QL block with 150 mg of ropivacaine may be safe. However, immediate transfer to the ward after QL block should be avoided, because the ropivacaine peak was observed around 30 to 60 minutes after the QL block [9].

There were a few randomized trials for the QL block. Murouchi et al. compared the intramuscular QL block with the lateral TAP block for laparoscopic surgery. Compared with the TAP block, QL block resulted in a widespread and long-lasting analgesic effect after laparoscopic ovarian surgery [9]. Blanco et al. compared the spinal anesthesia in addition to either the anterior or posterior QL block versus using only spinal anesthesia for caesarean sections [3]. The QL block after caesarean section was effective and provided satisfactory analgesia in combination with a typical postoperative analgesic regimen. In addition, Blanco et al. also compared the posterior QL block with the TAP block, where the posterior QL block was found more effective in reducing morphine consumption and demands than TAP block up to 48 hours postoperatively [14]. The QL block needs to be compared with other modalities to further prove its superiority and safety against others, such as epidural analgesia or the rectus sheath block.

In the present circumstances, the posterior, lateral, and intramuscular QL blocks are an effective analgesic method for abdominal surgery, particularly effective for lower abdominal surgery [3, 18]. From the viewpoint of safety and technique (Table 1), the intramuscular QL block may be an easier QL block for novice. Compared with these QL blocks, the anterior QL block may be an effective analgesia for the lower extremity surgery as well as the abdominal surgery.

There were no studies reporting complications after the QL block. Compared with the TAP block, some QL blocks are deep nerve blocks. Therefore, we must watch sites for infection, blood hematoma, and organ injuries [25, 26]. In particular, the anterior QL block is a deeper nerve block compared with the lateral and posterior QL block. From described above, consensus for the indications regarding each QL block should be achieved as soon as possible and our review could provide a comprehensive suggestion ready on the way.

## 8. Conclusion

The QL block is an effective analgesic tool for abdominal surgery and perhaps lower extremity. However, the best approach needs further validation and should be tailored to fit specific surgery whenever possible. Therefore, this review aims to make clear criteria to assess the performance of each QL block in comprehensive aspects, which facilitates future study design, provides the most updated knowledge, and contributes to the advances of techniques in deep regional blocks.

## Figures and Tables

**Table 1:** Multidimensional comparison regarding different approaches.

Approach	Analgesia	Technique	Safety	Reference
Anterior (subcostal)	T10 to L4 (T6-7 to L1-2)	Difficult	Not dangerous	Børglum et al. [8] (Elsharkawy [13])
Posterior	T7 to L1	Not easy	Safe	Blanco et al. [3]
Lateral	T7 to L1	Not easy	Not dangerous	Blanco et al. [3]
Intramuscular	T7 to T12	Easy	Safe	Murouchi et al. [8, 9]
